# Delay Patterns and Associated Factors Among Gastric Cancer Patients Visiting Tertiary Hospital in Ethiopia

**DOI:** 10.1002/cnr2.70209

**Published:** 2025-04-28

**Authors:** Tsegab Alemayehu, Semira Abdelmenan, Hailu Wondimu, Segni Kejela, Firaol Dandena, Tesfahun Ali, Zewdu Abadi, Zekarias Seifu

**Affiliations:** ^1^ School of Medicine College of Health Science, Addis Ababa University Addis Ababa Ethiopia; ^2^ Addis Continental Institute of Public Health Addis Ababa Ethiopia; ^3^ CURE Children's Hospital Ethiopia Addis Ababa Ethiopia

**Keywords:** diagnosis delay, Ethiopia, gastric cancer, patient delay, treatment delay

## Abstract

**Background:**

Gastric cancer ranks sixth in terms of incidence and fifth in terms of mortality in the world. It is also the fifth most frequent cancer in Ethiopia. In developed countries such as Japan, the diagnosis of gastric cancer is made early and has a better prognosis, but in developing countries like Ethiopia, the majority of patients present late in the advanced state. This study assessed delay patterns and associated factors among gastric cancer patients in Tikur Anbessa Specialized Hospital in Ethiopia.

**Methods:**

A single‐center cross‐sectional study was conducted on 64 gastric cancer patients on follow‐up from February 2021 to March 2023. The main outcome measures are the mean length of total delay, patient delay, diagnosis delay, and treatment delay. SPSS software version 26 and the Mann–Whitney statistical test were used to verify associations between the time intervals of access to treatment and socioeconomic factors, clinical variables, and patient‐reported reasons, adopting a 0.05 significance level.

**Results:**

In this study, the mean length of patient delay was 106 (SD = 142) days, the diagnosis delay was 318 (SD = 370) days, and the treatment delay was 43 (SD = 43) days. The average length of the total delay between symptom onset and definitive treatment was 467.4 (SD = 396.3) days. The greater length of patient delay in this study was correlated with lack of awareness (*p* < 0.001), search for traditional alternatives (*p* value 0.02), rural residence (*p* = 0.05), and economic hindrances (*p* = 0.01), and diagnosis delay was correlated with misdiagnosis (*p* < 0.001).

**Conclusion and Recommendation:**

Delays among gastric cancer patients in this study are much greater than those seen in other low‐income countries. Patient delay and diagnosis delay have a lion's share in the breakdown of the delays in our setup. Lack of awareness, the search for traditional alternatives, economic hindrances, and misdiagnosis were associated factors for delays. We recommend training primary healthcare providers regarding early signs of gastric cancer and integrating community‐based public health interventions to increase awareness of cancer and early health‐seeking behaviors. Along with increasing oncologic centers both by numbers and by quality of services.

**Abbreviations**
AAU: Addis Ababa University
ACIPH: Addis Continental Institute of Public Health
IRB: Institutional Review Board
MOH: Ministry of Health
TASH: Tikur Anbessa Specialized Hospital

## 1 Introduction

Gastric cancer is the third most frequent cause of cancer‐related death worldwide, with an estimated yearly mortality of 700,000 fatalities, placing it as a prominent illness that ranks fifth internationally [1]. It is the second most common cancer in Asian countries, and in Ethiopia, the literature puts gastric cancer variably; one reported it ninth among all cancers by incidence, and the GLOBOCAN report puts it as the fifth most common cancer in Ethiopia with incidence cases of 2580 in 2019 [2, 3]. Cancer incidence and mortality are primarily estimated by using nationwide cancer registries. However, Ethiopia has only one population‐based cancer registry, which is located in Addis Ababa; this makes the conclusion and discussion of nationwide incidence and mortality of cancer very challenging.

Early detection and treatment are crucial factors for improving cancer outcomes. Early diagnosis of gastric cancer results in better survival rates because it offers the opportunity to treat the disease when survival rates are higher [3, 4]. In countries with high incidences of gastric cancer like Japan and South Korea, nationwide screening programs with endoscopy have significantly improved the proportion of gastric cancer detected at an early stage and reduced mortality, with a 5‐year survival rate of over 90%. In other developed countries like the United States of America, early detection of the tumor is possible when all patients with new onset dyspepsia undergo upper gastrointestinal endoscopy [4, 5, 6].

In developing countries, challenges such as inadequate hospital infrastructure, limited healthcare resources, organizational deficiencies, and geographic barriers contribute to delays in cancer treatment. Patient‐related factors, including a lack of awareness about cancer risk factors, symptoms, and prognosis among patients, families, and healthcare professionals, financial barriers to accessing diagnosis and treatment, and a shortage of specialized oncology healthcare providers, contribute to delayed cancer treatment [7, 8, 9]. This is also true for Ethiopia [10].

Previous studies on delay among cancer patients mainly categorized the delay into three categories: primary delay or patient delay, diagnosis delay or secondary delay, and tertiary delay or treatment delay [8, 11, 12]. Primary delay or patient delay is defined as the duration between the onset of symptoms and the first presentation to the clinician. It may occur when the patient fails to recognize symptoms and seek medical attention, and even early symptoms are often treated by patients with medications such as those for peptic acid disease. There is no universally accepted cutoff point of time length for patient delay for gastric cancer thus far, but for colorectal cancer, it is 3 months, with 3 months or more defined as prolonged patient delay [13]. Diagnosis delays or secondary delay is the time after the first visit to a primary care provider until a biopsy‐proven diagnosis is made, which can be due to healthcare professionals not recognizing symptoms as serious, limited access to health services, initial negative endoscopy, delays in investigations, and misdiagnosis. Treatment delay or tertiary delay is the delay after a biopsy‐proven diagnosis is made until definitive care is given, which can be either surgery or commencement of chemotherapy or radiotherapy [14].

Most patients in developing countries present late with advanced gastric cancer, leading to high mortality and low 5‐year survival ranging from 30% to 40%. Delayed presentation, diagnosis, and treatment intervals pose challenges in managing these patients [3, 9, 14, 15].

Earlier studies in the 90s from the United Kingdom reported that the median delay among gastric cancer patients from the onset of symptoms to diagnosis was 22 weeks, and the delay by the patient after the onset of symptoms before seeking medical help was 4 weeks [15]. A recent study from Brazil by Mauro et al. found that the average length of delay from symptom onset to primary healthcare request was 471.3 days, and the average time between diagnosis and treatment commencement was 180.9 days [12]. Another Iranian study showed that the median total delay from the beginning of symptoms until surgery was 96 days, and the median patient delay (from first symptom to presentation to general practitioner) was determined to be eight days [16].

Studies on delay length among gastric cancer patients in Africa and Ethiopia are scarce. This study aims to identify the length of time intervals in these three categories of delay and associated factors in Tikur Anbessa Specialized Hospital (TASH) to take necessary measures.

## 2 Methods

### 2.1 Study Area and Design

A cross‐sectional study was conducted which included 64 biopsy‐proven gastric cancer patients who had definitive treatment from 20 February 2021 to 25 March 2023, at Tikur Anbessa Specialized Hospital (TASH). Information collected includes patient demographics such as age, sex, address, occupation, and education, as well as cancer‐specific information such as presenting symptoms, duration of symptoms, time interval from first symptom to diagnosis and treatment, family history of similar disease, and a probable reason for the delays. From previous studies in the same setting, age was categorized into above 45 years and below 45 years, considering the demography and the median age of gastric cancer patients; we also did the same [3, 6]. Main outcome measures are the length of total delay (time from first symptom onset to treatment), mean length of time from symptom onset to first healthcare visit (patient delay), from first healthcare presentation until diagnosis is made (diagnosis delay), and from diagnosis to the initiation of definitive treatment (treatment delay).

Incidentally diagnosed gastric cancer patients or those who are asymptomatic at the time of diagnosis are considered to have no patient delay; however, there was no observed asymptomatic patient in our context. Pairwise deletion was used in dealing with missing values to conserve available data from participants. However, during correlation analysis, cases (participants) with complete data for each variable were included.

TASH is a tertiary hospital with more than 700 beds located in Addis Ababa. It was established in 1964; the hospital provides different medical care services and serves as the main referral hospital for oncology services all over the country. On average, it gives service to close to 5500 new cancer cases a year [10].

### 2.2 Inclusion Criteria

Patients with a diagnosis of biopsy‐proven gastric cancer from 20 February 2021 to 25 March 2023, who were over 18 years of age and had undergone definitive treatment at TASH with surgical treatment, chemotherapy, or radiotherapy, which could be curative or palliative.

### 2.3 Exclusion Criteria

The study excluded patients who were hospitalized due to the recurrence of the disease.

Patients with clinical conditions that could limit their understanding or ability to respond to the interview questions.

Patients with a diagnosis of gastric cancer but insufficient information or incomplete clinical evidence for the diagnosis and treatment records.

Patients who were treated at TASH with a diagnosis of gastric cancer between 20 February 2021 and 25 March 2023, but were not alive during the data collection period (confirmation of death was obtained from their medical record if documented, or information obtained by telephone call for those not documented).

### 2.4 Data Collection Method and Tool

Data were collected from the medical chart and electronic medical record of each patient. Additional data were obtained using a structured interviewer‐administered questionnaire. The main outcome measures are the total delay and the delays related to patients themselves, from first healthcare presentation until diagnosis is made and from diagnosis to the initiation of definitive treatment.

### 2.5 Data Quality Assurance

A pretested data abstraction tool was used to extract data from the medical records of eligible participants, and the data was collected by one general practitioner and two junior surgical residents. The principal investigator provided half‐day training to data collectors on the tool, interview technique, ethical issues, participant rights, and confidentiality. The final version of the questionnaire was translated into Amharic and used for data collection, and the medical record chart and electronic medical record file were reviewed and filled out in data abstraction form. This is in order to cross‐check the data, and if information is missing from one source, it will be abstracted from the other source if it is available. The remaining data was obtained using a structured interviewer‐administered questionnaire. Data collection was closely monitored, and the principal investigator ensured the consistency and completeness of the data daily by cross‐checking the collected data with medical charts and electronic medical records. This study defined the date of first symptom as the first time the patient noticed or recognized the major symptom associated with stomach cancer. The date of the first healthcare visit was defined as the first time a patient visited/consulted with a healthcare practitioner after recognizing the first stomach cancer symptom. During interviews, several calendars and events (such as holidays) were used to aid in recall of the dates. Patients who could not recall the exact dates were given the choice of recalling months and weeks and the mid‐date of the week was chosen. However, the patient's medical record was used to determine the date of diagnosis confirmation.

### 2.6 Data Management and Analysis

Data was collected using the Kobo Toolbox application on a tablet, and the electronic data was then exported to SPSS software version 26.0 for data management and analysis. Before the initiation of analysis, the collected data was managed through data cleaning, transformation, and cross‐referencing of inconsistencies. Frequencies and percentages were obtained for each categorical variable and means and medians were obtained for continuous variables.

After checking normality distribution by the Shapiro–Wilk test of data, a nonparametric Mann–Whitney U test was used to verify associations between the time intervals of access to treatment and socioeconomic factors, clinical variables, and patient‐reported reasons for delay, adopting a 0.05 significance level.

## 3 Ethical Considerations

Before beginning the study, the Ethical Review Board of the Addis Continental Institute of Public Health and the Surgical Department of the Addis Ababa University College of Health Sciences (AAU CHS) provided ethical approval with a reference number of ACIPH‐MPH/059/15. This investigation was carried out in conformity with the Helsinki Declaration, the Ethiopian National Research Ethics Guidelines, and institutional research ethics policies. Prior to data collection, all study participants provided informed consent.

## 4 Results

Sixty‐seven patients fulfilled the eligibility criteria among patients with a diagnosis of gastric cancer who received definitive treatment at TASH between 20 February 2021 and 25 March 2023. Among them, 64 patients gave consent for the study, with a response rate of 95.5%, and were interviewed.

### 4.1 Socio‐Demographic Characteristics of the Study Population

Of the 64 patients, 54.7% (*n* = 35) were females, and the average age of the patients was 50.3 years, with a range of minimum of 21 and maximum of 78 years and a standard deviation of 14.3. A total of 42.2% of patients came from rural areas, and 57.8% were urban residents; 43.8% did not complete primary education and 35.9% (*n* = 23) were currently unemployed. In this study, the median family monthly income of the participants was 5000 Ethiopian birr (See Table 1).

**TABLE 1:** Sociodemographic characteristics of gastric cancer patients at TASH.

Variables (*n* = 64)		Frequency/mean	Percentage/SD
Age in years		50.3	±14
Age group	≤ 45 years	25	39.1%
	> 45 years	39	60.9%
Sex	Female	35	54.7%
	Male	29	45.3%
Region	Addis Ababa	22	34.4%
	Dire Dawa	1	1.6%
	Oromia	22	34.4%
	Amhara	7	10.9%
	SNNPR	12	18.8%
Residence	Rural	27	42.2%
	Urban	37	57.8%
Marital status (*n* = 63)	Currently married	49	77.8%
	Currently unmarried	14	22.2%
Education	Primary or less education	28	43.8%
	Secondary or above education	36	56.2%
Employment	Employed	40	62.5%
	Unemployed	23	35.9%
	Not documented	1	1.6%

### 4.2 Clinical Characteristics of the Study Population

A total of 15.6% had comorbidities, with hypertension being the most common, and only 3.1% of the study population had a family history of gastric cancer (see Table 2). The majority of patients had multiple initial presenting symptoms: 93.8% (60) had weight loss, 87.5% had epigastric pain, 75% had anorexia, 57.8% had vomiting, 33.3% had abdominal swelling, 26.2% had early satiety, 14.1% had dysphagia, and 20.3% had hematemesis. Furthermore, 67.2% of patients were at Stage III and above at the time of diagnosis, and 82.8% were at Stages 3 and 4 at the time of definitive care (see Table 2). The histologic types identified were intestinal type comprising 56.3% and diffuse type 18.8%; the rest are mixed type and others like GIST (see Table 2). A total of 48.4% of patients offered definitive treatment with curative intent and 51.6% with palliative intent (see Table 2).

**TABLE 2:** Clinical characteristics of the study participants at TASH.

Variables (*n* = 64)		*n* (%)/median (IQR)
Family history	No	62 (96.9%)
	Yes	2 (3.1%)
Number of contacts to any medical personnel before diagnosis		5 (4.5)
Number of contacts to medical personnel after diagnosis		3 (2)
Facility	Private	25 (39.1%)
	Government	39 (60.9%)
Stage at diagnosis	Not documented	6 (9.4%)
	Stage I	3 (4.7%)
	Stage II	12 (18.8%)
	Stage III	22 (34.4%)
	Stage IV	21 (32.8%)
Stage at first definitive care	Not documented	2 (3.1%)
	Stage I	2 (3.1%)
	Stage II	7 (10.9%)
	Stage III	18 (28.1%)
	Stage IV	35 (54.7%)
Histologic type	Diffuse type	12 (18.8%)
	Intestinal type	36 (56.3%)
	Mixed	1 (1.6%)
	Other	15 (23.4%)
Definitive treatments offered	Curative	31 (48.4%)
	Palliative	33 (51.6%)
Type of surgery	None	10 (15.6%)
	Bypass procedure	21 (32.8%)
	Subtotal gastrectomy	10 (15.6%)
	Total gastrectomy	10 (15.6%)
	Wedge resection	13 (20.3%)
Chemotherapy	None	16 (25.0%)
	Adjuvant	30 (46.9%)
	Neoadjuvant	3 (4.7%)
	Palliative	15 (23.4%)
Radiotherapy	Adjuvant	1 (1.6%)
	None	63 (98.4%)

### 4.3 Patient‐Reported Reasons for Delay

**FIGURE 1:**
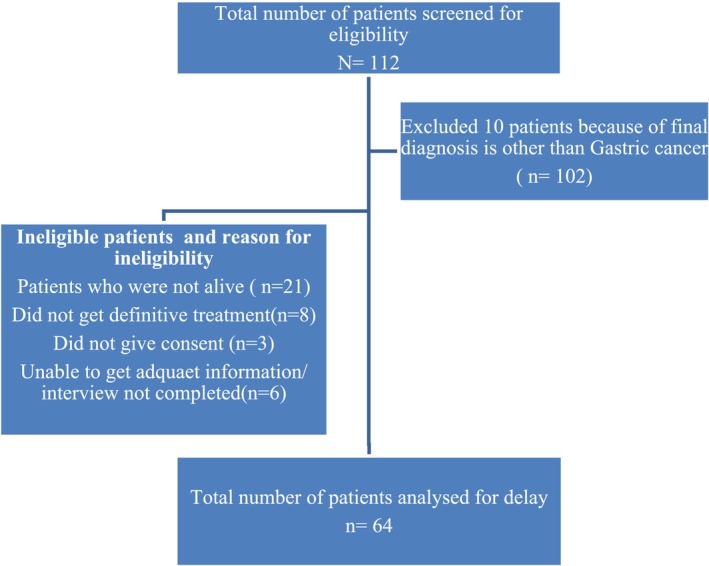
The flow charts of study participant selection.

**FIGURE 2:**
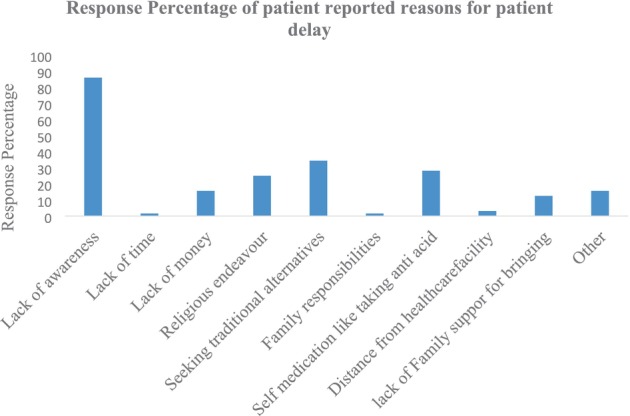
Reasons for patient delay of gastric cancer patients at TASH.

**FIGURE 3:**
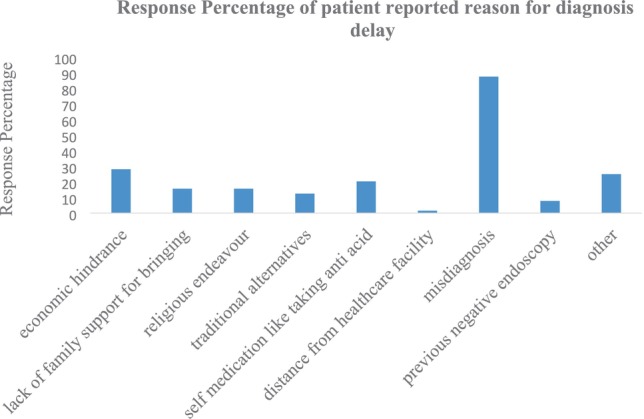
Reasons for diagnosis delay of gastric cancer patients at TASH.

**FIGURE 4:**
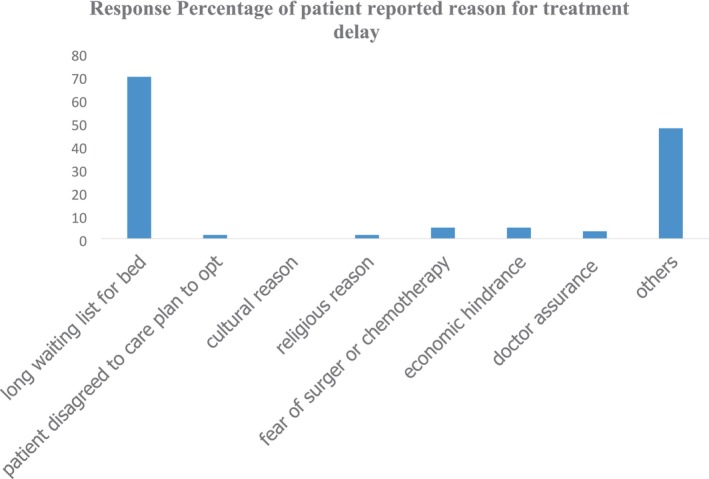
Reasons for treatment delay of gastric cancer patients at TASH.

Patients reported delays for a variety of reasons, the most common being lack of knowledge, looking for more conventional treatments, financial difficulties, and extended wait times (see Figures 1, 2, 3, 4) and Appendices A and B.

### 4.4 Delay Patterns Among Gastric Cancer Patients at TASH

The mean length of stay in days from symptom onset to first healthcare facility visit was 106 days, and the average length of time from first healthcare visit to biopsy‐proven diagnosis was 318 days, while the average length of stay after biopsy‐proven diagnosis until definitive treatment was made was 43 days. The average length of the total delay between symptom onset and definitive treatment was 467.42 days (See Table 3).

**TABLE 3:** Delay patterns from symptom onset to definitive care at TASH (in days).

Delay	Mean	Median	Min	Max	SD
Time from initial symptom to first healthcare facility visit in Days	106	60	7	720	142
Time from first healthcare presentation to diagnosis in Days	318	180	7	1440	370
Time from biopsy‐proven diagnosis to first definitive care in days	43	30	7	330	43
Total delay	467.4	300.0	97.0	1560.0	396.3

When we see factors that correlated with delays, the greater length of patient delay in this study was correlated with lack of awareness (*p* < 0.001), search for traditional alternatives (*p* value 0.02), rural residence (*p* = 0.05), and economic hindrances (*p* = 0.01), and diagnosis delay was correlated with misdiagnosis (*p* < 0.001) (See Table 4).

**TABLE 4:** Correlation of delay patterns with sociodemographic factors, clinical factors, and patient‐reported delay reasons in TASH.

Factors	Patient delay		Diagnosis delay		Treatment delay	
	Mean (SD)	*p*	Mean (SD)	*p*	Mean (SD)	*p*
Sociodemographic and clinical factors						
Age (≤ 45 vs. 45+ years)	105.5(163.6) vs. 106.0 (128.7)	0.4	432.6 (464.6) vs. 245.2 (277.4)	0.4	43.2 (26.3) vs. 43.3 (51.6)	0.42
Sex (male vs. female)	138.9(190.1) vs. 78.4(76.9)	0.56	275.69 (260.6) vs. 353.7 (441.9)	0.6	53.5 (57.4) vs. 34.7 (24.2)	0.02*
Residency (rural vs. urban)	136 (157.3) vs. 83.8 (127.5)	0.05*	235.6 (259.9) vs. 378.84 (426.9)	0.44	44.8 (26.8) vs. 42 (52.4)	0.15
Education (didn't complete primary education vs. Above or completed primary education)	90.4 (87) vs. 117.8 (173.6)	0.65	305.6 (356.4) vs. 328.3 (385.5)	0.97	43.1 (27.4) vs. 43.3 (43.3)	0.49
First diagnosis setup (government vs. private)	119.9 (141) vs. 83.8 (153.7)	0.053	281.7 (345.4) vs. 375.6 (406.7)	0.29	40.95 (24.1) vs. 46.8 (63)	0.37
Family history (yes vs. no)	120 (84.8) vs. 105.3 (143.9)	0.37	660 (763.6) vs. 307.37 (357.9)	0.42	60 (0.00) vs. 42.6 (43.9)	0.15
Stage at diagnosis (Stage I and II vs. Stage III and IV)	39.3 (42.7) vs. 129.6 (159.7)	< 0.01**	440 (435.1) vs. 289.7 (364.8)	0.14	38.1 (19.4) vs. 45.8 (50.8)	0.91
Physician contact before diagnosis (≤ 5 vs. > 5)	138.5 (168.8) vs. 68.8 (93.7)	< 0.01**	150.2 (115.4) vs. 509 (460.4)	< 0.01**	34.2 (20.7) vs. 53.5 (58)	0.06
Patient‐reported reasons						
Lack of awareness (yes vs. no)	120 (148.5) vs. 18.9 (17.6)	< 0.01**	296.3 (362.6) vs. 453.3 (410.3)	0.23	44.5 (46.3) vs. 35.7 (14.1)	0.87
Traditional alternatives (yes vs. no)	151 (167.7) vs. 82.12 (122.2)	0.02*	315 (393.7) vs. 320.2 (362.3)	0.51	46.9 (27.6) vs. 41.3 (49.7)	0.09
Self‐medication (yes vs. no)	93.6 (164.2) vs. 110.6 (134.1)	0.2	398.3 (420.7) vs. 287.1 (348.6)	0.14	60.3 (70.9) vs. 36.5 (23.6)	0.053
Economic hindrance (yes vs. no)	51.9 (79.8) vs. 126.9 (155.7)	0.01*	455 (431.1) vs. 264.9 (333.7)	0.02*	61.8 (73.4) vs. 36 (19.8)	0.21
Misdiagnosis (yes vs. no)	106.98 (150.5) vs. 97.5 (59.5)	0.27	352.5 (381.8) vs. 79.63 (118.5)	< 0.01**	45.3 (45.3) vs. 28.9 (20.8)	0.15
Long waiting list (yes vs. no)	105.4 (131.6) vs. 110.7 (170)	0.62	319.9 (387.6) vs. 320.5 (347)	0.69	52.8 (48.9) vs. 22.7 (11.7)	< 0.01**

*Note:* *Significance of association, **Corresponds to *P* value less than 0.01.

## 5 Discussion

This study explored the delay patterns among gastric cancer patients in tertiary hospitals in Ethiopia. The findings of this study showed the mean length of primary delay (patient delay) is 106 days, that of secondary delay (diagnosis delay) is 318 days, and the mean length of the treatment delay is 43 days. The average length of total delay between symptom onset and definitive treatment is 467.4 days (see Table 3).

Gastric cancer in Ethiopia has consistently been shown to be associated with advanced stage at diagnosis [3, 6]; concordant reports have been published from other low‐income countries [4, 16]. In this study, the proportion of Stage 1 and 2 disease at the time of diagnosis is only 23.4%. In countries with high incidences of gastric cancer like Japan, nationwide screening programs with endoscopy have significantly improved the proportion of gastric cancer detected at an early stage and reduced mortality, with a year survival rate of over 90%. In other developed countries like the United States of America, early detection of the tumor is possible when all patients with new onset dyspepsia undergo upper gastrointestinal endoscopy [4, 5, 6].

In Ethiopia, a lack of national screening programs, a limited number of endoscopy services, and a lack of awareness about the disease among the general population and even healthcare workers, together with the inherent nature of the disease, have contributed to the delay in diagnosis, resulting in advanced stage at presentation in developing countries [5, 10, 17].

### 5.1 Selected Socio‐Demographic and Clinical Factors

In this research, there was a slight preponderance of female patients with a male‐to‐female ratio of 1:1.2, which is contrary to two previous studies done by Hailu et al. and an earlier study by Johnson O et al. in the same hospital, which reported male preponderance, with a more recent report of a male‐to‐female ratio of 1.3:1, and other African countries reported the same result [3, 5, 6] This study result aligns with the general population makeup of the country, and the recent comparative study revealed that the sex difference was almost negligible under 45 years of age, while the relative difference maximized in the older age group, especially 65–69 [18]. A recent Korean study on the trend of incidence of gastric cancer showed that the age group 40–59 showed an increasing trend, especially in that the trend was more pronounced in females [19]. This is supported by the finding that this study's population mean age was 50.3 years, with 39.1% having an age below 45 years. In this study, being male is correlated with treatment delay; this might be due to males with gastric cancer being older and comorbidities being more common with old age, which might require preoperative optimization that prolongs their treatment compared to their female counterparts (see Table 4).

The proportion of rural residents in this study was 42.2%, and it is associated with greater delays in the presentation(see Table 4); this might be due to rural patients having significant geographic barriers with difficult transportation and distances to healthcare facilities, in addition to the limited number of health facilities in the rural area and the fact that many people must walk to healthcare centers. Furthermore, the majority of rural patients are illiterate with poor health‐seeking behaviors [10]. On the other hand, we hypothesized that not completing at least primary school might be associated with greater delays, but this did not happen in this study.

In our study, most of the study participants reported mixed symptoms during initial presentation, with weight loss and epigastric pain being the most commonly reported symptoms. This is in line with the previous study on the same hospital and several others, including one from New Mulango Hospital in Uganda by Ibingira CB [3, 20] which are symptoms of more advanced disease. Probably this is due to this patient having recall bias regarding which symptoms started first because of the retrospective nature of this study and the insidious nature of associated symptoms.

### 5.2 Factors Associated With Patient Delay

In this study, the mean length of patient delay is 106 days (22.7% of the total delay) and the median length is 60 days. This time interval is relatively lower than some other reports from low‐income countries; for example, 129 days in India. Another recent study in northern Brazil reported a mean patient delay of 471.3 days, which mainly attributed this delay to the use of traditional herbal medicine, while the Iranian study reported a mean length of this delay of 15.1 days [12, 16, 21]. Patient delay in this study was attributed to the search for traditional alternatives, lack of awareness, and economic hindrances. In high‐income countries, this delay is substantially different; for example, in England, it was 4 weeks [15].

The occurrence of a long time interval between the onset of symptoms and the first healthcare facility visit was correlated with being a rural resident, having stage III and IV disease at the time of diagnosis, and having five or fewer contacts with a physician before diagnosis was made (see Table 4). Lack of awareness (*p* < 0.001), search for traditional alternatives (*p* = 0.02), rural residence (*p* = 0.05), and economic hindrances (*p* = 0.01) were significantly correlated with patient‐reported reasons for patient delay (see Table 4). In this study, the occurrence of greater length of patient delay is correlated with having Stage III and IV disease at the time of diagnosis, which aligns with similar previous studies done elsewhere. These findings indicate that patients may not be fully aware of the symptoms of gastric cancer, or even if patients find these symptoms worrisome due to geographic barriers and economic hindrances, they do not seek health services early but rather prefer traditional alternatives of treatment. Therefore, raising awareness in the community regarding gastric cancer alarm symptoms is paramount in reducing the length of patient delay.

### 5.3 Factors Associated With Diagnosis Delay

The mean length of diagnosis delay in this study is 318 days (68% of total delay) with a median length of 180 days; this is higher than reports from other low‐income countries.

A study from Nepal reported a mean length of diagnosis delay of 220 days, and an Iranian study reported 96.4 days. They divided this delay into general practitioner delay and pathologist delay [16, 22]. Similar previous research suggests that diagnosis delays can be attributed to misdiagnosis; the application of other diagnostic laboratory tests and their interpretation may also take time, as well as the impact of preexisting patient conditions. Initial symptomatic treatment with acid suppression, combined with previous negative endoscopic results, adds to the delay in diagnosis [12, 22, 23]. This report is similar to our findings. In our study, having more than five contacts with physicians before a diagnosis was made; economic hindrance and misdiagnosis were significantly correlated with patient‐reported reasons for diagnosis delay (see Table 4).

### 5.4 Factors Associated With Treatment Delay

The average length of treatment delay in this study is 43 days and the median length is 30 days; this result is lower when compared with other low‐income countries. M, F.B. Filho, et al. reported 180.9 days from Northern Brazil, Soniya et al. from Nepal reported 50 days, and in contrast, an Iranian report was 10.09 days. The reasons for this delay were the long waiting list for in‐patient beds, appointments for metastatic radiological workups, and other related structural limitations. Our study finding was also not different from this; long intervals of time from biopsy‐proven diagnosis to definitive treatment were correlated with being male sex and long waiting time for bed [8, 16, 22]. Cancer control programs recommend treatment intervals of 30 days between diagnosis and treatment and less than 90 days between symptom onset and treatment [24]. Our study findings revealed that we are lagging behind this recommendation.

## 6 Strengths and Limitations of the Study

Because the data was acquired retrospectively and stomach cancer symptoms are nonspecific, there was a risk of recall bias in determining which symptom appeared first, and not all important information from patients' charts was probably recovered, which may pose bias in the measurement of time intervals. The biggest limitation of our study is that we did not perform a survival analysis due to the design of the study and the small sample size. Survival estimates would have helped us to determine whether the delay was associated with a poor prognosis. However, the authors of this study recommend that future researchers consider a prospective multicentric longitudinal study for further survival analysis. Another flaw is that individuals with incomplete medical records were omitted, which would have caused selection bias during secondary data collection, and the study duration also involved the late COVID‐19 pandemic era; the policies due to behavioral change could have caused further delay.

Despite these constraints, we believe the findings are robust, and the time interval corresponds to what we encounter in the clinic on a daily basis. This study was conducted at TASH, the country's first and largest oncological center, which serves the majority of the country's population. Furthermore, it created baseline data for future researchers, allowing us to move forward with future programs involving patient, family, and healthcare worker education at various levels of healthcare services, as well as at the community level.

## 7 Conclusion and Recommendations

In this study, patient delay and diagnosis delay are major components of the total delay, accounting for 22.7% and 68% of the total delay, respectively. These delays are much higher than reported delays from low‐income countries, and lack of awareness, search for traditional alternatives, rural residence, economic hindrances, and misdiagnosis were significantly correlated factors with these delays. Therefore, measures must be taken at each step of the delay to provide quality healthcare and improve the outcome of the cancer interventions.

Authors recommend the adoption of guidelines that address misdiagnosis and early endoscopy consideration, especially in assessing patients with symptoms of dyspepsia in the context of acid suppression therapy and traditional treatment options, to reduce diagnosis delay. This could be achieved by strengthening and standardizing our referral system at the policy level. General and Teaching hospitals **s**hould train their primary healthcare givers regarding early signs of gastric cancer to address misdiagnosis, and these health officers and general practitioners should be more cautious, especially when they have patients who are coming repeatedly to their clinics for antacid and acid suppression drugs. Early endoscope consideration in high‐risk patients, especially those above 45 years old with dyspeptic symptoms, is highly recommended.

A comprehensive cancer treatment program that encompasses preventive measures, early detection, diagnosis, and treatment (including palliative care) and curative measures should be strengthened by the Federal Ministry of Health (FMOH). It is crucial to increase the number of oncologic centers and the caliber of services provided by increasing the healthcare budget at the policy level for noncommunicable disease prevention and treatment. The authors recommend addressing the primary delay by collaborating with the public media to increase knowledge of early signs of stomach cancer. It is crucial to take steps to increase health literacy and launch campaigns that encourage people to seek medical attention as soon as symptoms appear. Overall, long‐term community‐oriented interventional approaches are highly imperative.

The FMOH should endeavor to enhance the accessibility of endoscopic services by augmenting the count of establishments and fortifying and enhancing the current healthcare infrastructure to mitigate diagnosis and treatment delays. Researchers should do a larger multicenter study on a cohort of gastric cancer patients, as this will allow them to collect as much data as possible and perform survival analysis as well as delay patterns.

## Author Contributions

**Tsegab Alemayehu:** conceptualization, methodology, validation, formal analysis, investigation, and writing – original draft preparation. **Semira Abdelmenan:** methodology, formal analysis, writing – review and editing, and supervision. **Hailu Wondimu:** validation, writing – review and editing, and supervision. **Segni Kejela:** investigation and data curation. **Tesfahun Ali:** resources and data curation. **Zewdu Abadi:** data curation and resources. **Zekarias Seifu:** resources and data curation. **Firaol Dandena:** writing – original draft preparation and writing – review and editing. All the authors have read and approved the final manuscript.

## Ethics Statement

Before beginning the study, the Ethical Review Board of the Addis Continental Institute of Public Health and the Surgical Department of the Addis Ababa University College of Health Sciences (AAU CHS) provided ethical approval.

## Consent

The authors have nothing to report.

## Conflicts of Interest

The authors declare no conflicts of interest.

## Data Availability

The data that support the findings of this study are available from the corresponding author upon reasonable request.

## Appendix A

Data abstraction form.

**Table:**

SN	Question	Information	Skip
1.	Age of the patient		
2.	Medical record number		
3.	Sex	1. Female 2. Male	
4.	Stage at diagnosis (at presentation to TASH)	1. Stage I 2. Stage II 3. Stage III 4. Stage IV 5. Not documented	
5.	Stage at first definitive care	1. Stage I 2. Stage II 3. Stage III 4. Stage IV 5. Not documented	
6.	Histologic type	1. Intestinal type 2. Diffuse type 3. Mixed	
7.	Grade of tumor	1. Grade I 2. Grade II 3. Grade III 4. Unspecified	
8.	Treatments offered to patients presenting with gastric cancer	1. Curative 2. Palliative	
9.	Type of surgery if done	1. Total gastrectomy 2. Subtotal gastrectomy 3. Bypass procedure	
10.	Chemotherapy given	1. Neoadjuvant 2. Adjuvant 3. Palliative 4. None	

## Appendix B

Interview tool.

**Table:**

SN	Question	Response	Skip
1.	Medical record number		
2.	Regional state	1. Addis Ababa 2. Oromia 3. Amhara 4. SNNPR 5. Tigrai 6. Somali 7. Afar 8. Harari 9. Gambella 10. Benishangul 11. Diredawa	
3.	Residential area	1. Urban 2. Rural	
4.	Marital status	1. Married 2. Single 3. Widowed 4. Divorced	
5.	Occupation	1. Homemaker 2. Student 3. Retired 4. Civil servants 5. Business person 6. Unemployed 7. Other	
6.	Education level	1. Didn't complete primary education 2. Completed primary education 3. Completed secondary education 4. Completed college level	
5.	Comorbidities	1. Diabetes 2. Hypertension 3. HIV 4. Cardiac illness 5. Neurologic disorder 6. Other 7. None	
6.	Family history of gastric cancer	1. Yes 2. No	
7.	Monthly INCOME of family in ETB		
8.	Initial symptoms (multiple answers possible)	1. Anorexia 2. Weight loss 3. Malaise 4. Nausea 5. Vomiting 6. Indigestion 7. Epigastric pain 8. Shortness of breath 9. Dysphagia 10. Malaena 11. Hematemesis 12. Constipation 13. Other 14. Abdominal swelling	
9.	Number of contacts to any medical personnel before diagnosis is made		
10.	Number of contact to medical personal after diagnosis made		
11.	Time from initial symptom to first healthcare facility in months		
12.	Time from first healthcare presentation to diagnosis in months		
13.	Time from biopsy proven diagnosis to first definitive care in months		
14.	Reason for delay from symptom onset to first visit (multiple answers possible)	1. Lack of awareness 2. Lack of time 3. Lack of money 4. Religious endeavor 5. Traditional alternatives 6. Self‐medication like taking antacids, etc. 7. Distance from healthcare facility 8. Economic hindrance 9. Lack of family support (for bringing her/him in) 10. Family responsibilities 11. Other	
15.	Reason for delay from first presentation to diagnosis	1. Economic hindrance 2. Lack of family support (for bringing her/him in) 3. Religious endeavor 4. Traditional alternatives 5. Self‐medication like taking antacids, etc. 6. Distance from healthcare facility 7. Misdiagnosis Previous negative endoscopy 8. Other	
16.	Reason for delay from diagnosis to definitive care	1. Long waiting list 2. Patient disagreed with the care plan and opted for alternative routes (like religious) 3. Cultural reason 4. Religious reason 5. Fear of surgery or chemotherapy 6. Economic hindrance to undergoing surgery 7. Doctor assurance (i.e., doctor assured the patient that he/she may receive cancer treatment after some time period or no major problem) 8. Others	
17.	Type of service requested at the beginning of symptoms	1. Private 2. Public	
